# EFFECTS OF HIGHER- AND LOWER-INTENSITY EXERCISE ON FITNESS, COGNITION, MOTOR FUNCTION, AND QUALITY OF LIFE IN ADULTS WITH TRAUMATIC BRAIN INJURY

**DOI:** 10.2340/jrm-cc.v8.44345

**Published:** 2025-11-17

**Authors:** Monica E. Soliman, Cris Zampieri, Lisa M.K. Chin, Diane L. Damiano

**Affiliations:** Rehabilitation Medicine Department, National Institutes of Health, Clinical Center, Bethesda, MD, USA

**Keywords:** aerobic exercise, balance, mobility, rehabilitation

## Abstract

**Objective:**

To assess the effects of higher-intensity aerobic training (AET) and lower-intensity rapid-resisted exercise training (RET) on fitness, cognition, balance, mobility, and quality of life in sedentary adults with chronic traumatic brain injury.

**Design:**

Participants were randomized to AET, RET, or waitlist control later randomized to AET or RET.

**Participants:**

Nine adults, 25 to 65 years, completed elliptical training (AET = 4; RET = 5). Follow-up data were available for 4 AET and 2 RET.

**Methods:**

Exercise groups trained for 12 weeks. Outcomes were assessed at 0, 12, and 24 weeks.

**Results:**

Main effects from exercise included improvements in the Brief Visuospatial Memory Test, Limits of Stability excursion, fast elliptical cadence, and self-reported cognitive abilities. Improved fitness was related strongly to improved memory, balance, and quality of life. Similar fitness gains across groups indicate high individual variability in response to exercise intensity. Continuing to exercise during follow-up was associated with more cognitive benefits.

**Conclusion:**

Exercise had positive effects on multiple aspects of functioning well after traumatic brain injury and should be advocated. Differences based on exercise intensity were not identified in this small sample. Inconsistent recommendations across studies on optimal exercise parameters are likely obscured by individual differences, suggesting a personalized approach is warranted.

## LAY ABSTRACT

The aim was to evaluate the effects of exercise on adults with a traumatic brain injury who could walk but were generally inactive. We randomly assigned nine participants to high-intensity training exercise or lower-intensity exercise focusing on increasing movement speed. Before and after a 12-week supervised training program and after a 12-week follow-up, we measured their ability to think quickly and recall things. We assessed physical abilities like fitness levels, how fast they walked, and how good their balance was, and we also collected ratings of how satisfied they were with their life and abilities. No differences were found between exercise types; however, when groups were combined, positive effects on thinking, moving, and satisfaction were observed. Those with greater fitness after training, which interestingly was not related to training intensity, had greater improvement. In conclusion, regular exercise at a safe level should be advocated for people with a brain injury.

Traumatic Brain Injury (TBI) affects 3.2 to 5.3 million Americans with nearly 125,000 per year having long-term impairments (1, 2). Recovery from the initial injury is modest, with 25% declining further over time (3). Even after rehabilitation, difficulties persist with many experiencing lower quality of life (QOL), increased depression, higher social isolation, and reduced employment (4).

Exercise is highly recommended to improve neurocognitive function after TBI (5, 6) and shown to aid neural recovery and alleviate secondary symptoms (7). Studies have demonstrated that exercise can reduce cerebral inflammation, stimulate neurogenesis, and reduce the negative cascade causing neurodegeneration, anxiety, learning, and memory deficits (8–10). A recent study using an animal TBI model concluded that beneficial effects of exercise on memory were dependent on exercise intensity and sex (11).

A previous systematic review concluded that exercise had generally positive effects on fitness, gait, and balance in adults with chronic TBI (12), while acknowledging limited data quality and quantity. A more recent review (13) found that virtual-reality balance training was more effective than standard balance training, and physical activity improved fatigue and QOL post-training but not at follow-up. No conclusions could be made about the most beneficial training type or intensity (13), highlighting the need for more research to develop exercise guidelines in TBI. Another review of randomized controlled trials (14) showed that light-to-moderate exercise improved QOL when performed regularly for 6+ weeks, with shorter durations showing no benefits. Authors cautioned that high intensity may exacerbate symptoms, and exercise effectiveness may attenuate with greater chronicity (14).

Studies have compared different exercise intensities with disparate findings. Wu et al. (6) compared low- and high-intensity treadmill exercise in rats, concluding that low-intensity improved, whereas high intensity worsened, spatial memory. In contrast, sedentary overweight males performing moderate-intensity continuous training or high-intensity interval training displayed similar improvements in brain-derived neurotrophic factor (BDNF) levels and cognition (15). Further confounding the picture, a randomized control trial (16) showed that high-intensity step training improved gait speed, aerobic capacity, efficiency, and cognition more than stepping practice.

We conducted two pilot exercise studies in participants with chronic TBI on different training intensities: a low-to-moderate intensity in-home rapid-resisted elliptical training (17) and a supervised aerobic training (AET) protocol (18). While each demonstrated benefits, differences in sample size, duration, and outcomes made comparisons across interventions untenable.

Our primary objective was to compare the effects of two 12-week elliptical exercise programs, higher-intensity AET and lower-intensity rapid-resisted training (RET), on fitness, cognition, mobility, balance, and QOL in adults with chronic TBI, to relate changes across functional domains, and to compare changes to a follow-up period when individuals were encouraged to continue exercising on their own. In contrast to previous exercise studies that evaluated outcomes in only one or a few domains, we decided here to more comprehensively assess and relate measures across multiple key functional domains.

## MATERIALS AND METHODS

### Ethical approvals

This study was a registered RCT (clinicaltrials.gov# NCT02504866 on July 22, 2015), with institutional review: National Institutes of Health Clinical Center [15-CC-0164], Uniformed Services University of the Health Sciences [CNRM-53-3897, CN1-10-01NIH], and George Mason University [776565]. Recruitment for this study began on 9/15/2016 and was stopped early due to the COVID-19 pandemic. A written-informed consent was obtained from all participants prior to any study procedures or assessments.

### Participants

Participants were recruited from the greater Washington DC metropolitan region. Inclusion criteria were as follows: 1) adults (aged 18 to 79 inclusive); 2) diagnosed with a non-penetrating TBI that occurred at least 12 months prior to enrollment; 3) physically inactive as identified by a physician; 4) able to safely stand and walk independently; 5) able to comply with the study protocol; 6) fluent in English and able to provide informed consent. Exclusion criteria were as follows: 1) history of exercise intolerance; 2) history of cardiac disease, pulmonary disease (other than controlled, non-exercise-induced asthma), uncontrolled diabetes, or uncontrolled hypertension; 3) on medication that influences aerobic capacity or treadmill performance (e.g. beta blockers and antiretroviral therapy); 4) active substance abuse; 5) injury or medical condition affecting motor function or the ability to perform the assessments or exercise (e.g. vestibulopathy); 6) unable to refrain from smoking at least 4 h prior to exercise testing; 7) pregnancy; 8) BMI>40 kg/m^2^ (exceeds limit of the treadmill, elliptical machine, and MRI scanner); 9) planning to modify medication/therapy aimed at improving mood, cognitive function, or motor function during the study.

### Study design

Once enrolled, participants performed a baseline assessment of physical fitness, neuropsychology, mobility, balance, symptoms, and QOL. Participants were then randomized to one of three groups: 1) waitlist control, 2) aerobic exercise group (AET), or 3) rapid-resisted exercise group (RET) for 12 weeks. After 12 weeks, assessments were repeated. Participants in the control group were then randomized to either AET or RET, while those in the exercise groups ceased formal training. All returned at 24 weeks for their final assessment.

Randomization was performed by an independent randomizer, not associated with the protocol, using randomized blocks. Investigators were given sealed envelopes containing the group assignment, to be opened in sequential order, after the participant has completed the baseline assessment.

Based on sample size calculations, this RCT aimed to have 20 participants in each of the three groups. Due to low recruitment rates and the pandemic, we are only able to report on the participants who completed trial participation before study closure.

### Exercise intervention

All exercise sessions were supervised by research staff and performed on an elliptical machine 3 times per week for 12 weeks, for 30 min (plus 15 min of warm-up or cool-down) at the NIH Clinical Center and followed one of two protocols:

*Aerobic Exercise Training (AET).* The higher-intensity sessions were performed at a target heart rate (HR) range of 70% to 80% of the heart rate reserve (HRR), where HRR=0.70 to 0.80 × (peak HR – resting HR) + resting HR. Peak HR was based on the Cardiopulmonary Exercise Test (CPET) performed prior to intervention. Progression focused on increasing elliptical resistance over speed to maintain the target HR, as tolerated.

*Rapid-resisted Exercise Training (RET).* The lower-intensity sessions emphasized rapid speed. Participants progressively increased speed over time with gradual increases in resistance once their speed target was met and were guided to maintain a HR below the AET group.

During exercise sessions, participants wore a heart rate monitor (Polar H10) and reported perceived exertion using the Borg Category-Ratio Scale (0–10 rating). Elliptical resistance and cadence (in revolutions per minute, rpm) were also recorded.

### Outcome measures

Physical fitness was assessed during the CPET. Briefly, pulmonary gas exchange was measured breath-by-breath using a computerized metabolic cart (CardiO2 Ultima, MGC Diagnostics Corp, St. Paul, MN) during incremental treadmill exercise to a participant’s tolerance limit. A 12-lead ECG was utilized to monitor safety and record HR data. Measures included peak O_2_ consumption (VO_2_ averaged over the final 20s), test duration, HR, and treadmill work rate at test completion. Peak VO_2_ was also expressed as a percent predicted for a sedentary person, based on sex, weight, and age (19), with ≤ 84% of predicted indicating low exercise tolerance. The respiratory exchange ratio (RER) was used to assess peak effort on the CPET as >1.10.

Neuropsychological measures included the following: 1) Trail Making Tests (TMT) A and B (primary outcome), timed tests that measures processing speed and attention (TMT-A) and executive function (TMT-B), expressed as T-scores adjusted by age and education with cognitive impairment indicated as ≤ 37 (20); 2) Delis-Kaplan Executive Function System (D-KEFS) Sorting Test free sorting condition, a measure of executive function with scaled scores ≤ 7 indicative of potential impairment (21); 3) Brief Visual Memory Test-Revised (BVMT-R), a non-verbal learning and memory test, with T-scores < 37 considered impairment in TBI (22); 4) California Verbal Learning Test (CVLT-II) that assesses verbal learning and memory, with free recall T-scores < 40 suggesting memory impairment (23). For all measures, higher T-scores, scaled or standard scores, indicated better cognitive functioning.

Mobility measures included gait speed in cm/s and elliptical cadence in rpm. For gait speed, participants walked twice across a 4.8-meter-long instrumented walkway (GAITRite, CIR Systems Inc., Sparta, NJ) at their freely chosen speed and as fast as possible without running. Elliptical cadence, performed at a self-selected and fastest pace without resistance, assessed reciprocal coordination.

Balance was assessed with the NeuroCom Smart Equitest system (previously Natus, Inc, Middleton, WI) and included the Sensory Organization Test (SOT), Motor Control Test (MCT), and Limits of Stability (LOS). Briefly, the SOT measures sensory postural control, where the individual attempts to stand still for 20 s under different sensory conditions (somatosensory, visual, and vestibular). SOT variables were reported as ratios. The MCT tests the latency (msec) of automatic postural reactions where the individual is to remain standing upright during sudden platform translations that prompt activation of long loop automatic postural reflexes. The LOS measures volitional control of posture where one moves their center of mass to their base of support limits in eight directions, yielding composite scores for reaction time (s), movement velocity (˚/s), endpoint and maximal excursions, and directional control (%). For SOT and LOS measures, except reaction time, higher values indicate better performance.

Self-reported questionnaires assessed fatigue, sleep, depression, and other common TBI-related symptoms. The Fatigue Severity Scale (FSS) averages 9-items, with values ≥ 4 associated with significant severity (24). The Pittsburgh Sleep Quality Index (PSQI) includes 7 components for a global sleep score, with scores > 5 indicating dysfunction (25). The Beck Depression Inventory-II (BDI-II) measures severity of depressive symptoms, with a total score of ≥ 19 or ≥35 indicating depression among those with mild or moderate/severe TBI, respectively (26). The Brief Symptom Inventory 18 (BSI-18) sums the severity of psychological symptoms in three domains (somatization, depression, and anxiety) for a Global Severity Index, with T-scores ≥ 63 indicating greater distress (27). The Neurobehavioral Symptom Inventory (NSI) assesses the presence and severity of 22 symptoms, yielding a total score and one for each subcategory: Vestibular, Somatosensory, Cognitive, and Affective (28). For all questionnaires, higher values reflect greater severity.

Satisfaction and QOL specific to the TBI population were assessed by: 1) Satisfaction with Life Scale (SWLS) with scores < 20 indicating significant dissatisfaction (29); 2) TBI-QOL with composite index scores (mean = 100, standard deviation = 15) reported for physical, emotional, cognitive, and social health domains, and an overall global QOL score (30); 3) Quality of Life after Brain Injury (QOLIBRI) for domains of Cognition, Self, Daily Life and Autonomy, Social Relationships, Emotional, Physical, and overall QOL, with scaled scores < 60 for overall QOLIBRI indicate poor health-related QOL (31). Lower scores on all indicate poorer satisfaction with life or QOL.

Participant’s exercise habits were queried through monthly phone calls during the 12-week follow-up period. Those responding “Yes” to having exercised were asked about the exercise frequency (i.e. number of times/week), duration (i.e. minutes per session), and type performed that month. The metabolic equivalent (MET, in MET×min/week) was estimated as the product of frequency, duration, and MET value (32, 33) for the reported exercise.

### Statistical analyses

Independent t-tests were used to compare exercise parameters between AET and RET groups. General Linear Models (GLMs) with repeated measures were used to compare the effects of AET vs RET on each outcome measure, with time as the within-subject and group as the between-subject factors. Associations among changes in fitness parameters and across functional domains were explored with Spearman’s Rank Order correlation procedures. Paired t-tests were used to compare changes after exercise with groups combined to those after the follow-up period in the same participants. Cohen’s d effect sizes were calculated and interpreted as small (0.20), moderate (0.50), and large (0.80). Correlation coefficients (ρ) were interpreted as follows: weak (0.30 or less), moderate (0.40–0.60), and strong (>0.70). All analyses were performed with IBM SPSS Statistics software (version 29.0.2.0). Significance was set at p < 0.05 (two-tailed).

## RESULTS

### Participants

Twenty participants were consented into the study. Five met exclusion criteria, while 4 withdrew before randomization. Of the 11 participants randomized, 4 were assigned to AET, 3 to RET, and 4 to CON, with 2 withdrawn, 1 during training, and 1 during the 12-week assessment for cardiac-related concerns. The remaining 2 were later randomized to and completed RET for a total of 9 who exercised (4 AET and 5 RET; see Table I for participant demographics). Mean age was 51 (18) and 44 (11) years for AET and RET, respectively. Six had follow-up data (4 AET and 2 RET) for the analysis comparing the exercise and follow-up periods.

**Table I:** Demographic characteristics of participants

Code	Age (years)	Group	Sex	TBI severity^a^	Time from injury (years)	Race	BMI (kg/m^2^)	Mechanism of injury
A-1	25	AET-F	F	Mild	10.6	White	22.4	Direct impact-blow to head
A-2	65	AET-F	F	Moderate	3.5	Asian	22.1	Acceleration/deceleration
A-3	53	AET-F	M	Severe	1.9	White	23.2	Fall (height >1 meter)
A-4	60	AET-F	F	Severe	20.3	White	22.3	Acceleration/deceleration
R-1	40	RET-F	M	Moderate	13.2	Multiple	22.9	Blast
R-2	61	RET	F	Moderate	1.2	White	22.4	Fall (ground floor)
R-3	49	RET-F	M	Severe	20.2	White	25.5	Aircraft Accident
R-4	37	CON-RET	M	Moderate	9.2	Multiple	23.9	Direct Impact-Head against object
R-5	32	CON-RET	M	Moderate	5.4	White	25.8	Fall (height >1 meter)

A or AET: higher-intensity aerobic exercise training; R or RET: lower-intensity rapid-resisted exercise training; AET-F or RET-F: exercise intervention then follow-up; CON-RET: waitlist control then RET; F: female; M: male.

a TBI severity by VA/DoD clinical practice guidelines for the management of concussion-mild traumatic brain injury, 2016. Available from: https://www.va.gov/covidtraining/docs/mTBICPGFullCPG50821816.pdf

### Pre-intervention assessments

Tables SI–SIV show the scores prior to exercise. All participants gave good effort on the CPET (RER > 1.10) and averaged 98 (17)% of expected cardiorespiratory fitness. Only 3 failed to reach 84% of peak predicted VO_2_ indicating normal exercise tolerance in 67% of participants. Free gait speed was normal (34). Up to four participants demonstrated significant cognitive impairments for any one of the given assessments, and at most, only one participant reported significant fatigue, psychological symptoms, sleep difficulties, or poor satisfaction and QOL on the respective questionnaires. Abnormal scores were more prevalent on balance tests, particularly the LOS endpoint and maximal excursion on which 6 of 9 participants scored below normal.

### Exercise training parameters

Table II shows the training parameters for the AET and RET groups. High attendance was observed in both groups (mean of 92% to 93%). AET participants had higher average heart rate (HR), ratings of perceived exertion (RPE), and elliptical resistance during sessions, yet cadence was not different between groups.

**Table II:** Comparison between AET and RET groups in training parameters

Exercise training parameters	AET	RET	Mean difference (95% CI)	*p*-value	Cohen’s d
Cadence (rpm)	46.65 (6.92)	38.36 (9.24)	8.28 (−4.92 to 21.49)	0.182	1.00
Resistance (level)	7.36 (4.55)	1.00 (0.00)	6.36 (1.64 to 11.08)	**0.015** *	2.14
HR (bpm)	147.26 (14.11)	104.28 (12.89)	42.98 (21.69 to 64.28)	**0.002** *	3.20
RPE	3.71 (0.88)	1.79 (0.98)	1.92 (0.44 to 3.41)	**0.018** *	2.05

AET: higher-intensity aerobic exercise training; RET: lower-intensity rapid-resisted exercise training; rpm: revolutions per minute; HR: heart rate; bpm: beats/min; RPE: Rating of Perceived Exertion.

* Significant at *p*<0.05.

One serious adverse event (cardiac related) occurred during an AET session, and the participant was withdrawn. Other adverse events such as musculoskeletal (e.g. back or knee pain), cardiac (e.g. arrhythmias), nervous system (e.g. headache), endocrine (i.e. hypoglycemia), and psychiatric symptoms (pre-existing condition) were reported but considered minor or low risk, and all were followed to resolution.

### Comparison for AET vs RET groups on outcomes

Mean age did not differ significantly between groups (*p* = 0.50). Table III shows the effects of training across groups on all measures with a statistically significant result (*p* < 0.05) or non-significant trend (*p* < 0.10). All other variables are shown in Table SV. Notably, several outcomes demonstrated a positive main effect for time (changes due to exercise with groups combined), with no significant main effect for group or group-by-time interactions. Significant improvements in learning and visuospatial memory were seen in the BVMT-R immediate and delayed recall T-scores and on the QOLIBRI cognition score. Reciprocal coordination as assessed by fast elliptical cadence was significantly higher post-training. Balance significantly improved on the LOS maximal excursion composite score post-training.

**Table III:** General Linear Model to assess exercise effects pre- and post-training

Outcomes	Group	Pre-exercise (mean [SD])	Post-exercise (mean [SD])	*p*-value
**Cardiorespiratory fitness**				
Time to peak exercise (seconds)	AET	654.75 (123.45)	707.78 (164.40)	**Time = 0.071** Group = 0.286 Interaction = 0.508
	RET	592.20 (40.01)	619.00 (53.63)	
**Cognitive assessments**				
BVMT-R: Immediate Recall (T-score)	AET	43.50 (16.90)	48.50 (20.07)	time = 0.039* group = 0.415 interaction = 0.365
	RET	49.40 (17.05)	60.60 (10.55)	
BVMT-R: Delayed Recall (T-score)	AET	44.75 (19.62)	47.75 (16.13)	time = 0.048* group = 0.639 interaction = 0.154
	RET	43.20 (15.55)	58.40 (7.54)	
D-KEFS: Correct Sorts (Scale score)	AET	13.75 (2.99)	12.25 (0.96)	time = 0.790 group = 0.456 **interaction = 0.095**
	RET	11.00 (3.16)	13.00 (1.22)	
**Mobility assessments**				
Elliptical Cadence: Fast (rpm)	AET	84.76 (9.20)	89.69 (7.65)	time = 0.023* group = 0.507 interaction = 0.204
	RET	87.05 (20.99)	101.19 (17.14)	
Gait Velocity: Regular walking (cm/s)	AET	146.65 (16.74)	152.83 (11.69)	**time = 0.089** group = 0.189 interaction = 0.523
	RET	129.96 (11.18)	142.52 (20.20)	
Gait Velocity: Fast walking (cm/s)	AET	226.00 (17.68)	222.58 (10.57)	time = 0.176 group = 0.762 **interaction = 0.078**
	RET	219.76 (38.38)	241.70 (41.93)	
**Balance assessments**				
LOS: Composite Movement Velocity (˚/s)	AET	3.6 (2.09)	4.38 (2.29)	**time = 0.058** group = 0.907 interaction = 0.617
	RET	3.54 (1.91)	4.78 (2.53)	
LOS: Composite Directional Control (%)	AET	80.00 (5.35)	84.25 (3.50)	**time = 0.080** group = 0.615 interaction = 0.587
	RET	78.40 (9.04)	80.80 (9.31)	
LOS: Composite Max Excursion (%)	AET	79.00 (10.20)	88.50 (13.18)	time = 0.042* group = 0.918 interaction = 0.814
	RET	79.00 (15.05)	86.80 (12.54)	
SOT: Somatosensory	AET	98.50 (2.38)	96.75 (1.50)	**time = 0.099** group = 0.979 interaction = 0.774
	RET	98.80 (2.17)	96.40 (2.30)	
**Questionnaires**				
QOLIBRI: Cognition (Scale score)	AET	62.50 (39.82)	68.75 (37.95)	time = 0.031* Group = 0.702 Interaction = 0.503

Results for group (AET or RET) and time (Pre- to Post-training) main effects and group by time interaction.

SD: standard deviation; AET: higher-intensity aerobic exercise training; RET: lower-intensity rapid-resisted exercise training; BVMT-R: Brief Visuospatial Memory Test-Revised; D-KEFS: Delis-Kaplan Executive Function System sorting test; SOT: Sensory Organization Test; LOS: limits of stability; QOLIBRI: Quality of Life after Brain Injury. Bold indicates *p*<0.10.

* Significant at *p*<0.05.

### Relationships between changes in outcomes after exercise training

**Fig. 1:**
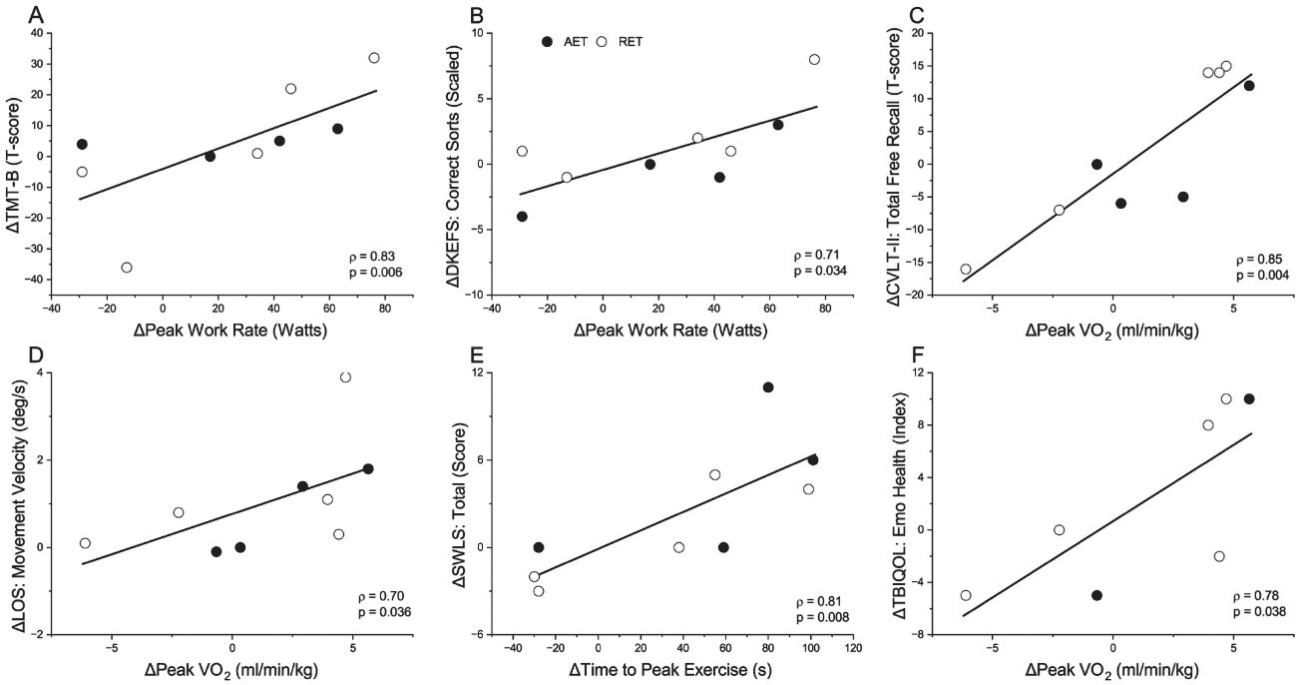
Strong significant positive (p<0.05) Spearman correlations among changes in fitness parameters and neuropsychological (A-C), balance (D), and quality of life (E, F) outcomes: (A) peak work rate with Trail Making Test B (TMT-B); (B) peak work rate with Delis-Kaplan Executive Function System sorting test (DKEFS); (C) peak oxygen consumption (VO_2_) with California Verbal Learning Test (CVLT-II) free recall; (D) peak VO_2_ with Limits of Stability (LOS) movement velocity; (E) time to peak VO_2_ with Satisfaction with Life Scale (SWLS) total score; (F) peak VO_2_ with TBI Quality of Life (TBIQOL) emotional health subscale. Each symbol represents a change score (post- minus pre-training) for an individual in the higher-intensity aerobic exercise training (AET, black) or lower-intensity rapid-resisted exercise training (RET, white) group. Incomplete data for TBI-QOL on 2 participants. Those with greater fitness gains tended to have greater improvements in functional domains.

**Fig. 2:**
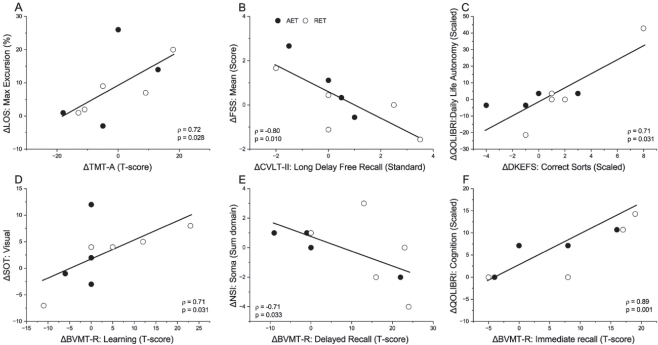
Strong significant (*p*<0.05) Spearman correlations between changes in cognitive outcomes and balance measures (A, D), symptom severity (B, E), and quality of life outcomes (C, F). (A) Trail Making Test A (TMT-A) and Limits of Stability (LOS) maximal excursion; (B) California Verbal Learning Test (CVLT-II) delayed recall and Fatigue Severity Scale (FSS) symptoms; (C) Delis-Kaplan Executive Function System sorting test (D-KEFS) and Quality of Life after Brain Injury (QOLIBRI) daily life and autonomy; (D) Brief Visual Memory Test-Revised (BVMT-R) learning and Sensory Organization Test (SOT) vision; (E) BVMT-R delayed recall and Neurobehavioral Symptom Inventory (NSI) somatosensory symptoms; (F) BVMT-R immediate recall and QOLIBRI cognition. Each symbol represents a change score (post- minus pre-training) for an individual in the higher-intensity aerobic exercise training (AET, black) or lower-intensity rapid-resisted exercise training (RET, white) group. Those with greater cognitive gains tended to have greater improvements in other domains.

**Fig. 3:**
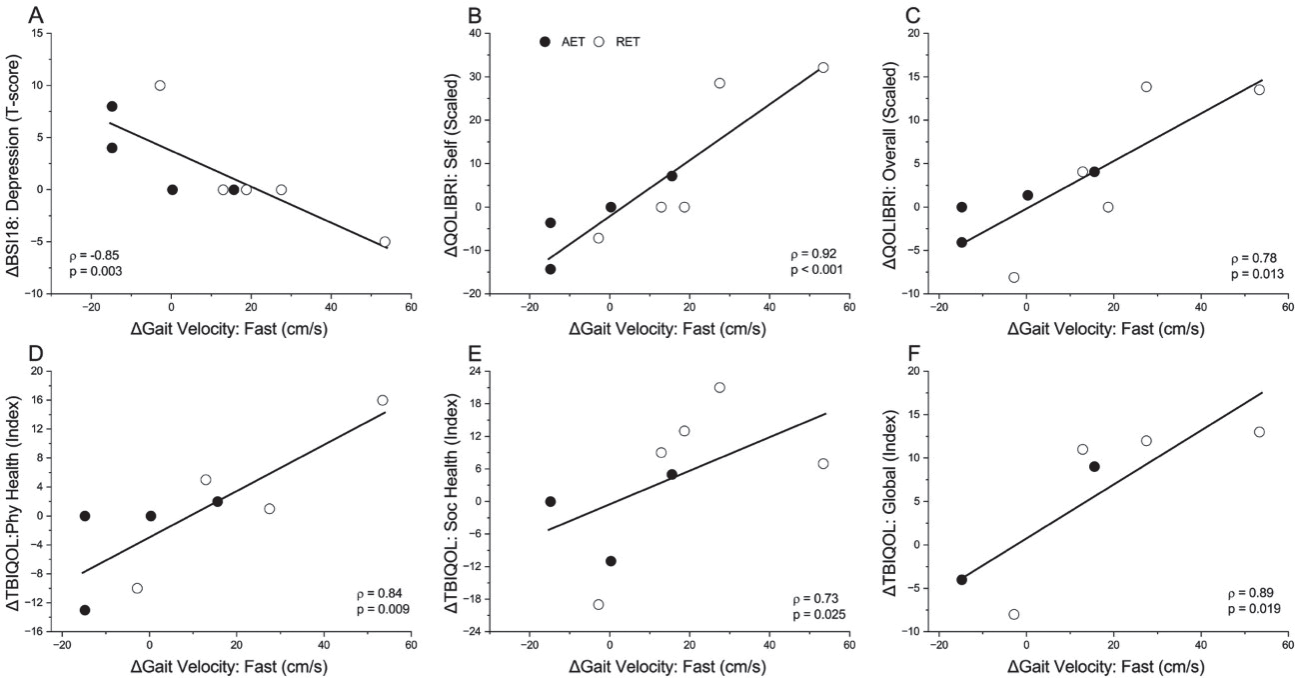
Strong significant (*p*<0.05) Spearman correlations between changes in fast gait speed and (A) Brief Symptom Inventory (BSI-18) depression severity; (B) Quality of Life after Brain Injury (QOLIBRI) self; (C) QOLIBRI overall score; (D) TBI Quality of Life (TBIQOL) physical health; (E) TBIQOL social health; (F) TBIQOL global score. Each symbol represents a change score (post- minus pre-training) for an individual in the higher-intensity aerobic exercise training (AET, black) or lower-intensity rapid-resisted exercise training (RET, white) group. Incomplete data for TBI-QOL on 1 to 3 participants. Those with greater gait speed gains tended to have greater improvements in symptom severity and quality of life.

Fig. 1 shows the significant strong associations between exercise-related changes. Greater changes in fitness indicators correlated with improvements in executive functioning (1A and 1B) and verbal memory (1C). Strong positive correlations were observed between fitness gains and LOS movement velocity (1D), and satisfaction with life and emotional health (1E and 1F). Fig. 2 shows the significant strong associations across functional domains. TMT-A improvements were positively related to those on the LOS maximal excursion (2A), while greater changes in the BVMT-R learning scores were related to greater SOT vision scores (2D). Improvements in delayed memory were related to decreased fatigue severity (2B) and fewer somatosensory symptoms (2E), while improvements in executive function on D-KEFS and immediate memory on BVMT-R correlated with better QOL for daily life/autonomy (2C) and cognition (2F), respectively. Finally, Fig. 3 shows a strong negative correlation between fast gait speed and depression severity on BSI-18 (3A), and strong positive correlations between increased fast gait speed and improved QOL (3B-F).

### Durability of changes following exercise training

Table IV displays the estimated exercise performed (in MET×min/week) during follow-up, which questioned the continuation of exercise, frequency, and duration and type of exercise if performed. Two participants (1 AET, 1 RET) exercised for all 3 months, while others only exercised for 1 or 2 months.

**Table IV:** Individual changes during follow-up

Code	Month 1	Month 2	Month 3	Mean (SD)
A-1	1102.5	1102.5	1732.5	1312.5 (363.7)
A-2	0	0	580.5	193.5 (335.2)
A-3	0	0	450	150 (259.8)
A-4	954	1166	0	706.7 (621.1)
R-1	735	1155	735	875 (242.5)
R-3	344.5	0	0	114.8 (198.9)

MET: Metabolic equivalent of task; A: randomized to higher-intensity aerobic exercise training; R: randomized to or performed lower-intensity rapid-resisted exercise training; SD: standard deviation.

Estimated MET×min/week from the monthly phone follow-up logs post-visit 2.

**Fig. 4:**
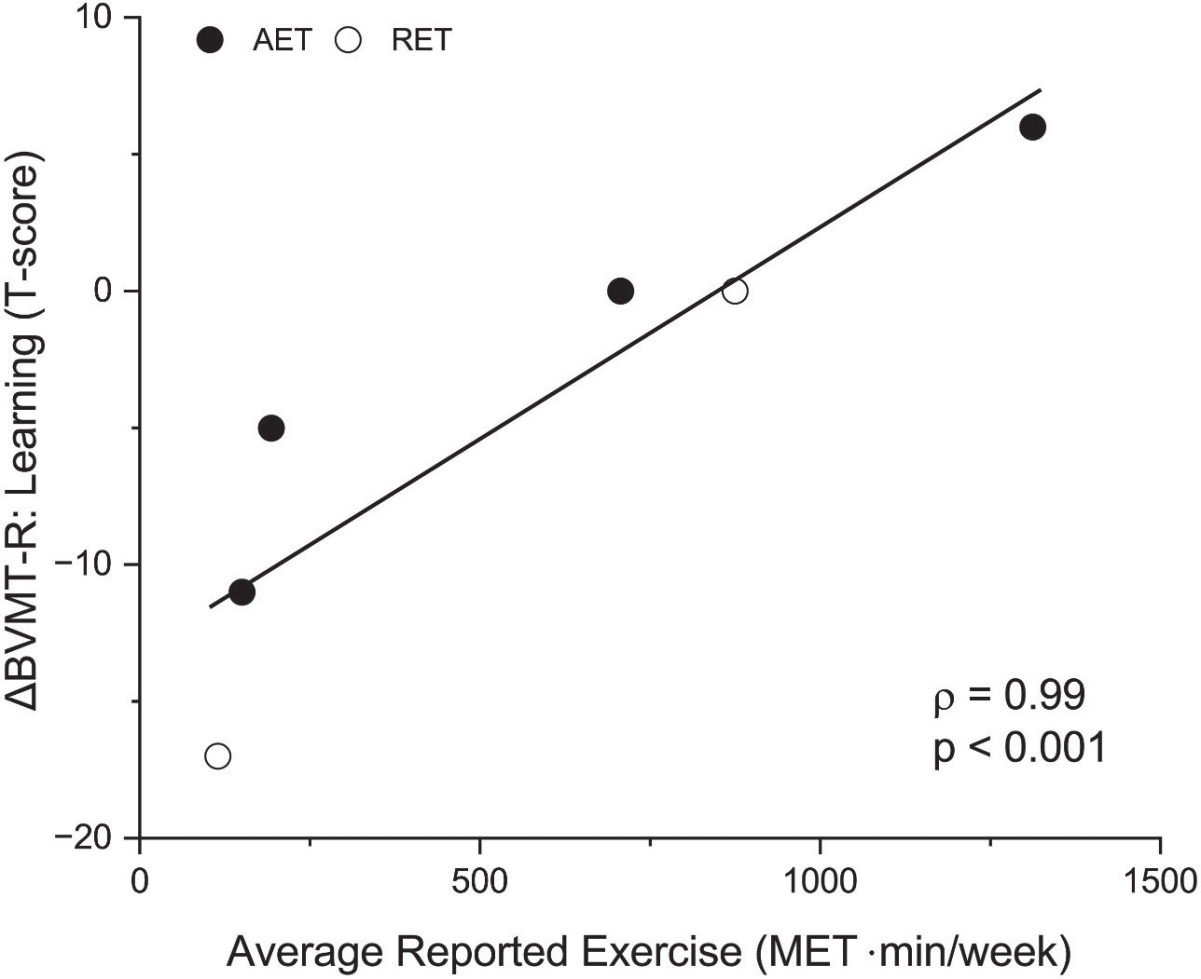
Correlation among reported exercise (estimated as metabolic equivalent, MET, in MET×min/week) and changes in visuospatial learning on the BVMT-R during follow-up. Each circle represents an individual in the higher-intensity aerobic exercise training (AET, black) or lower-intensity rapid-resisted exercise training (RET, white) group.

Results comparing change scores during training vs follow-up periods are shown in Table SVI. No significant changes indicate effects were maintained. The estimated MET×min/week strongly and directly related to BVMT-R learning T-score changes during follow-up (Fig. 4; ρ=0.99, *p*<0.001), suggesting continuation of exercise may further improve specific aspects of cognitive function.

## DISCUSSION

Our initial objective to compare the effects of higher-intensity aerobic exercise training (AET), lower-intensity RET, and a waitlist control group on physical fitness, cognition, mobility, balance, and QOL outcomes in ambulatory adults with chronic TBI was not achieved due to the low number of recruited participants. However, when exercise groups were combined, improvements across functional domains supported the benefits of exercise in TBI, as seen in healthy adults and other disorders. Those with greater fitness gains tended to experience greater improvements.

### Baseline function

Participants exhibited better than expected baseline cardiorespiratory fitness. Only three participants reported substantial fatigue, poor sleep, and/or QOL issues, with four participants achieving normal performance on all neuropsychological measures. This contrasted with previous studies reporting high rates of exercise intolerance (35), fatigue and disruptive sleep (36), and cognitive dysfunction (37) in individuals with TBI. One exception was voluntary balance where 6 of 9 participants demonstrated significant deficits, consistent with previous findings (38). The high level of functioning in our participants likely attenuated responses to training. Furthermore, participants were on average 9.5 years from their initial TBI, and large changes resulting from exercise would not be expected (14).

### Outcomes of exercise interventions

Exercise groups here intentionally differed in intensity, with higher heart rate, RPE, and elliptical resistance in AET. Attendance was similarly very high across groups. All four participants in AET exercised at vigorous intensity, while two of five participants in RET trained at moderate intensity and three at light to very light intensity. The three exercising at the lowest intensities showed collective improvement in cardiorespiratory fitness (+ 4.3 (0.4) ml/min/kg), higher than the AET group (+ 2.1 (2.8) ml/min/kg), while the two exercising at moderate intensity showed worsened fitness levels (− 4.2 (2.8) ml/min/kg) after training. These findings underscore the variability in results reported across studies attempting to offer guidance on optimal training parameters despite multiple studies, RCTs, and systematic/meta-analyses performed thus far. The variability in rate and degree of recovery after TBI may be attributable to the complex and heterogeneous nature of these injuries and premorbid clinical, psychological, and social factors (39). Our findings point to individualized, rather than group level recommendations and may further support the importance of promoting exercise among those with TBI not rooted, per se, on intensity.

The primary outcome here for comparing the exercise protocols was a cognitive one; however, our small sample yielded limited power to detect group differences. When groups were combined, significant changes in multiple domains were found. Specifically, BVMT-R improved for both immediate and delayed recall. As mental processing speed is a significant predictor of BVMT-R scores due to the speed of item presentation in the test (40), these results are consistent with the rationale for the rapid training cadence in the RET group (and matched by the AET group): movement speed may influence cognitive processing speed (17). A recent systematic review on the effects of exercise on cognitive recovery in TBI concluded that existing but limited evidence supported improvements in visuospatial and delayed memory, as well as processing speed (41). The authors further noted (41) that strong evidence of cognitive benefits from exercise in animal models of TBI and healthy adults has surprisingly not translated as robustly to adults with TBI, suggesting that TBI-specific symptoms such as fatigue, apathy, mobility, or balance impairments may limit exercise participation, which could potentially alleviate many of these. From a mechanistic standpoint, exercise promotes neurogenesis in the dentate gyrus of the hippocampus with the number of new neurons produced correlated with improvements in memory and retention (42). Evidence also supports enhanced functional connectivity from exercise, which directly relates to processing speed (43). Importantly, improved self-reported cognitive abilities on QOLIBRI mirrored the BVMT-R outcomes.

Despite a lack of consensus on exercise intensity in TBI, moderate-intensity exercise improved cognitive and physical function in a meta-analysis on older adults with dementia, with potentially similar benefits in other brain disorders such as TBI (44). A recent study compared moderate-intensity aerobic cycling to stretching and toning (45), measuring cognitive function and structural brain changes in individuals with memory impairment post-TBI. Although not statistically significant, large effect sizes (Cohen’s d > 1.4) were found for improvements in cognitive tasks related to processing speed and increased brain volume in the left hippocampus and right thalamus for the exercise group (45).

### Correlations across outcomes

Given the variability in responses across individuals with TBI, group mean changes are not sufficient to ascertain the potential benefits of exercise on a per person basis (46). Correlations link individual training responses to outcomes and across domains. Mean improvement in TMT-B, our primary outcome, was not demonstrated as hypothesized; however, only a third of our sample demonstrated deficits on TMT-B prior to training. Effects did emerge when correlating changes on TMT-B and other cognitive tests with changes in fitness and other functional domains. Increased peak VO_2_ and work rate were strongly and directly correlated with increases in TMT-B T-scores, D-KEFS card sorting score, and CVLT-II recall T-score, consistent with our previous study in TBI (47) and others (48, 49), implying that exercise type or intensity is less relevant than individual improvement in fitness for improving cognitive outcomes. Greater TMT-A scores also showed positive associations with improved LOS maximal excursion, endpoint excursion, and movement velocity, which is particularly notable since TMT-A measures processing speed. These task-specific associations across domains could indirectly support greater efficiency in brain pathways. Further insights incorporating biomarkers and imaging may shed more light on these relationships. Indeed, similar BDNF increases and cognitive improvement among overweight men training at different exercise intensities (15) also indicated that exercise type/intensity may matter less than the one most effective for a given individual.

### Follow-up

TBI is increasingly recognized as a chronic and evolving condition (50), although prognosis varies considerably with nearly one-fourth continuing to improve and another fourth experiencing decline (51). Prognosis worsens markedly for those with repetitive injuries (52). For those with milder brain injuries, decline is likely attributable to lifestyle factors before or post-injury, such as sedentary behavior (53). We specifically recruited physically inactive participants, yet more than a third demonstrated higher fitness than predicted for sedentary adults of similar age and sex. The follow-up period was probably too short to detect a significant decline in function and confounded by the recommendation to continue exercising, which participants performed to varying degrees. As a strong positive association was observed between improved BVMT-R and greater physical activity during follow-up, continuing to exercise may accrue further benefits on cognitive function.

### Adverse events

Several cardiac-related events (one serious) were encountered despite extensive exclusion criteria to minimize risks: thorough history and physical, and review of baseline ECG prior to exercise. All sessions were performed in a hospital with a qualified team. The risk of serious cardiac events (i.e. acute myocardial infarction) during or after vigorous-intensity exercise is greater in middle-aged and older adults compared to younger adults and higher still in those previously sedentary (54), which may represent many with chronic TBI. Current recommendations suggest a gradual phased approach to increasing exercise duration and intensity over several months in previously sedentary otherwise healthy individuals before engaging in vigorous exercise to help mitigate risks (54). Given the current lack of evidence supporting the superiority of higher-intensity exercise in TBI, the decision to pursue high intensity exercise should be carefully considered on an individual basis. Light to moderate exercise could be performed with fewer safety concerns and encouraged as a lifestyle choice for this more vulnerable population. While we chose a frequency of 3 times a week to balance achieving a training effect with the subject/resource burden, the literature suggests that more frequent sessions would be preferable (13).

### Limitations

We did not achieve our target enrollment; therefore, results were not sufficiently powered for detecting group differences. The low enrollment also created more of a contrast to the relatively large number of outcomes assessed here. Despite a large cohort of individuals with TBI participating in a natural history study in our department, recruitment from this or other local sources for our TBI exercise trials was challenging even prior to the pandemic, which halted recruitment for an extended period. Barriers may include TBI-related factors as mentioned earlier that limit engagement in exercise, difficulty with travel to NIH, and issues such as need to take off time from work. The absence of a sufficiently large control group also limited the ability to evaluate exercise effects compared to any change in function, positive or negative, in a group that was not exercising over the same time period.

### Conclusions

This relatively short-duration exercise program effectively enhanced certain motor and cognitive abilities in sedentary individuals with chronic TBI long after injury, reinforcing the importance of regular exercise in this population. However, the issue of optimal exercise intensity for TBI remains unclarified and may only be answerable on a more personalized basis.

## Supplementary Material


