# Supplementary material for: EFFECTS OF HIGHER- AND LOWER-INTENSITY EXERCISE ON FITNESS, COGNITION, MOTOR FUNCTION, AND QUALITY OF LIFE IN ADULTS WITH TRAUMATIC BRAIN INJURY
Source: J Rehabil Med Clin Commun. 2025 Nov 17;8:44345. doi: 10.2340/jrm-cc.v8.44345 (PMC12639367; doi:10.2340/jrm-cc.v8.44345)
Supplement: Supplementary file 1 [file JRMCC-8-44345-s1.pdf]

Supplementary material has been published as submitted. It has not been copyedited, or typeset by Journal of Rehabilitation Medicine – Clinical Communication

Table SI: Subject demographics, Cardiopulmonary Exercise Test (CPET) and gait assessment results before exercise intervention

**Demographics**				**CPET**		**Gait**
**Group**	**Sex**	**TBI Severity**	**Age (years)**	**RER**	**Peak VO_2 (% pred)**	**Regular Walk Velocity (cm/s)**
A-1	F	Mild	25	1.25	109	153.2
A-2	F	Moderate	65	1.23	111	123.3
A-3	M	Severe	53	1.43	***82***	162.5
A-4	F	Severe	60	1.14	112	147.6
R-1	M	Moderate	40	1.33	***79***	137.7
R-2	F	Moderate	61	1.25	123	139.2
R-3	M	Severe	49	1.38	99	117.0
R-4	M	Moderate	37	1.20	***73***	118.5
R-5	M	Moderate	32	1.45	88	137.4

Note: A=randomized to higher intensity aerobic exercise training (AET), R=randomized to or performed lower intensity rapid-resistive exercise training (RET); RER=respiratory exchange ratio, VO_2=O_2 consumption, pred=predicted. M = male, F = female. Bold and italic font indicates lower than predicted normal value.

Table SII. Results from neuropsychological tests before exercise intervention

Group ID	Neuropsychological Assessments						
	**TMT-A (T-score)**	**TMT-B (T-score)**	**D-KEFS: Correct Sorts (Scaled)**	**BVMT-R: Imm. recall (T-score)**	**BVMT-R: Learning (T-score)**	**BVMT-R: Delayed recall (T-score)**	**CVLT-II: Learning (T-score)**
A-1	40	46	13	41	46	***32***	***38***
A-2	58	54	13	52	62	62	62
A-3	52	43	18	60	46	61	72
A-4	56	***34***	11	***21***	***35***	***24***	47
R-1	39	***26***	10	44	41	***35***	42
R-2	51	42	12	60	41	53	58
R-3	66	76	13	48	63	44	51
R-4	62	***32***	***6***	***25***	52	***22***	***27***
R-5	64	72	14	70	***28***	62	72

Note: A=randomized to higher intensity aerobic exercise training (AET), R=randomized to or performed lower intensity rapid-resistive exercise training (RET); TMT-A=Trail Making Test A, TMT-B=Trail Making Test B; D-KEFS= Delis-Kaplan Executive Function System sorting test; BVMT-R= Brief Visual Memory Test-Revised; CVLT-II= California Verbal Learning Test. Bold and italic font indicates meeting cutoff for abnormal scores on respective assessments.

Table SIII. Balance test results before exercise intervention

Group ID	Balance Assessments										
	**SOT: Comp**	**SOT: Vest**	**SOT: Som**	**SOT: Vis**	**SOT: Pref**	**MCT: Comp (msec)**	**LOS: RT (sec)**	**LOS: EPE (%)**	**LOS: MVL (deg/sec)**	**LOS: MXE (%)**	**LOS: DCL (%)**
A-1	87	75	97	97	107	122	0.69	73	5.2	***79***	79
A-2	73	67	102	***74***	94	***160***	1.46	***53***	***1.6***	***75***	74
A-3	76	55	97	90	108	148	0.82	81	5.6	93	87
A-4	***64***	***43***	98	84	94	142	1.00	***38***	2.0	***69***	80
R-1	***51***	***9***	101	84	87	132	0.78	***64***	3.7	***74***	72
R-2	80	65	98	91	102	138	1.36	***50***	***1.2***	***67***	89
R-3	80	67	101	91	108	139	0.85	***38***	2.6	***65***	75
R-4	***67***	***17***	96	81	147	152	0.93	***62***	3.8	89	69
R-5	81	74	98	91	101	138	0.52	94	6.4	100	87

Note: A=randomized to higher intensity aerobic exercise training (AET), R=randomized to or performed lower intensity rapid-resistive exercise training (RET); SOT = Sensory Organization Test, comp = composite, vest = vestibular, som = somatosensory, vis = visual, pref = preference; MCT = motor control test; LOS = limits of stability, RT = composite reaction time, EPE = composite endpoint excursion, MVL = composite movement velocity, MXE = composite maximal excursion, DCL = directional control. Bold and italic font indicates lower than normal performance.

Table SIV. Results from self-reported questionnaires before exercise intervention

Group ID	Self-Reported Questionnaires							
	FSS: Mean (Score)	PSQI: Global (Score)	BDI-II: Total (Score)	BSI-18: Global (T-score)	NSI: Total (Score)	SWLS: Total (Score)	TBIQOL: Global (Percentile)	QOLIBRI: Overall (Score)
A-1	3.9	2	9	45	9	33	55	84
A-2	1.0	1	0	39	3	***18***	98	97
A-3	2.3	3	3	45	4	31	79	93
A-4	1.3	None	7	47	17	29	None	67
R-1	3.7	2	0	36	2	30	96	95
R-2	2.0	5	9	48	17	20	45	74
R-3	***5.3***	4	11	***65***	30	24	27	***53***
R-4	1.0	***10***	0	36	12	21	55	62
R-5	1.0	0	0	36	1	35	99	100

Note: A=randomized to higher intensity aerobic exercise training (AET), R=randomized to or performed lower intensity rapid-resistive exercise training (RET); FSS = Fatigue Severity Scale; PSQI = Pittsburgh Sleep Quality Index; BDI-II = Beck Depression Inventory-II; BSI-18 = Brief Symptom Inventory 18; NSI = Neurobehavioral Symptom Inventory; SWLS = Satisfaction with Life Scale; TBI-QOL = TBI-Quality of Life; QOLIBRI = Quality of Life after Brain Injury. Bold and italic font indicates meeting cutoff for abnormal scores on respective questionnaires.

Table SV. Comparison between exercise groups pre- and post- training including all variables

**Outcomes**	**Group**	**Pre-exercise (mean± SD)**	**Post-exercise (mean ± SD)**	**p-Value**
**Cardiorespiratory Fitness (n=9)**				
Relative VO_2 at Peak Exercise (mL/kg/min)	AET	30.02±4.99	32.08±7.26	time= 0.313<br>group= 0.477<br>int=0.700
	RET	33.10±2.65	34.05±6.12	
Work Rate at Peak Exercise (W)	AET	327.50±87.87	350.75±77.24	time= 0.144<br>group= 0.200<br>int=0.988
	RET	417.80±88.87	440.60±122.05	
Absolute VO_2 at Peak Exercise (L/min)	AET	2006.51±549.95	2132.30±621.20	time= 0.355<br>group= 0.277<br>int=0.780
	RET	2515.65±512.11	2584.49±767.67	
RER at Peak Exercise	AET	1.26±0.12	1.30±0.07	Time=0.888<br>Group= 0.792<br>int=0.320
	RET	1.32±0.10	1.27±0.14	
Heartrate at Peak Exercise (bpm)	AET	173.25±16.09	174.00±16.10	Time=0.898<br>Group= 0.947<br>int=0.898
	RET	174.60±25.58	174.60±23.82	
**Cognitive Assessments (n=9)**				
BVMT-R: Learning (T-score)	AET	47.25±11.12	45.75±11.73	time= 0.536<br>group= 0.865<br>int=0.305
	RET	45.00±13.17	50.80±14.34	
TMT-B (T-score)	AET	44.25±8.26	48.75±4.79	time= 0.605<br>group= 0.705<br>int= 0.903
	RET	49.60±23.04	52.40±27.74	
TMT-A (T-score)	AET	51.50±8.06	49.00±8.60	time= 0.752<br>group= 0.291<br>int= 0.819
	RET	56.40±11.33	56.00±11.40	
CVLT-II: Total Free Recall (T-score)	AET	54.75±15.17	55.00±13.09	time=0.620<br>group=0.787<br>int=0.661
	RET	20.00±16.90	54.00±19.01	
CVLT-II: Short Delay Free Recall (Standard score)	AET	0.25±1.32	-0.25±2.25	Time=0.825<br>Group=0.442<br>Int=0.211
	RET	-1.20±1.75	-0.50±1.32	
CVLT-II: Long Delay Free Recall (Standard score)	AET	-0.25±1.50	-0.25±2.25	Time=0.530<br>Group=0.569<br>Int=0.530
	RET	-1.30±2.20	-0.50±1.32	
**Mobility (n=9)**				
Elliptical Cadence: Self-selected (rpm)	AET	49.04±12.34	49.09±4.89	Time=0.472<br>Group=0.368<br>Int=0.465
	RET	46.27±10.73	40.73±10.69	
**Balance Assessments (n=9):**				
LOS: Reaction Time (s)	AET	0.99 ± 0.34	0.80±0.22	Time=0.254<br>Group=0.821<br>Int=0.580
	RET	0.89± 0.31	0.82±0.37	
LOS: End Point Excursion (%)	AET	61.25±19.47	68.75±20.11	Time=0.116

	RET	61.60±20.90	65.80±19.23	Group=0.923<br>Int=0.629
MCT: Composite (msec)	AET	143.00±15.87	144.75±15.90	Time=0.229<br>Group=0.870<br>Int=0.523
	RET	139.80±7.36	145.20±11.48	
SOT: Vestibular	AET	60.00±14.00	69.25±16.54	Time=0.224<br>Group=0.361<br>Int=0.852
	RET	46.40±30.80	58.80±19.69	
SOT: Visual	AET	86.25±9.74	88.75±4.86	Time=0.239<br>Group=0.694<br>Int=0.944
	RET	87.60±4.77	90.40±5.03	
SOT: Preference	AET	100.75±7.80	103.25±10.44	Time=0.799<br>Group=0.613<br>Int=0.476
	RET	109.00±22.59	103.80±10.11	
SOT: Composite	AET	75.00±9.49	79.00±5.72	Time=0.273<br>Group=0.627<br>Int=0.899
	RET	71.80±12.99	76.80±8.41	
**Questionnaires (n=9, unless noted otherwise):**				
FSS (Mean score)	AET	2.14±1.30	3.03±0.82	Time=0.408<br>Group=0.965<br>Int=0.296
	RET	2.60±1.88	2.49±1.40	
PSQI: Global Sleep (Total score); n=8	AET	2.00±1.00	3.33±1.53	Time=0.699<br>Group=0.655<br>Int=0.156
	RET	4.20±3.77	3.40±4.39	
BDI-II (Total Score)	AET	4.75±4.03	2.50±2.08	Time=0.523<br>Group=0.861<br>Int=0.368
	RET	4.00±5.52	4.40±6.80	
BSI-18: Somatization (T-Score)	AET	42.75±3.50	41.25±0.50	Time=0.456<br>Group=0.438<br>Int=0.215
	RET	46.20±9.96	46.60±10.85	
BSI-18: Depression (T-Score)	AET	40.25±0.50	43.25±3.95	Time=0.258<br>Group=0.396<br>Int=0.557
	RET	45.80±11.30	46.80±8.84	
BSI-18: Anxiety (T-Score)	AET	46.75±6.60	46.75±7.85	Time=0.525<br>Group=0.770<br>Int=0.525
	RET	47.20±11.23	43.40±5.86	
BSI-18: Global Severity Index (T-Score)	AET	44.00±3.46	43.00±6.93	Time=0.807<br>Group=0.890<br>Int=0.573
	RET	44.20±12.74	44.60±10.43	
NSI: Vestibular (Sum of domain)	AET	0.50±0.58	0.25±0.50	Time=0.292<br>Group=0.662<br>Int=0.292
	RET	0.80±1.79	0.80±1.79	
NSI: Somatosensory (Sum of domain)	AET	1.75±0.96	1.75±1.26	Time=0.798<br>Group=0.332<br>Int=0.798
	RET	3.60±3.78	3.20±2.77	
NSI: Cognitive (Sum of domain)	AET	3.00±2.83	2.75±3.10	Time=0.740<br>Group=0.696<br>Int=0.429
	RET	3.60±3.65	4.20±4.97	

NSI: Affective (Sum of domain)	AET	2.50±2.08	1.25±0.96	Time=0.547 Group=0.308 Int=0.547
	RET	4.40±4.28	4.40±5.13	
NSI: Total (Sum all items)	AET	8.25±6.40	6.50±5.80	Time=0.710 Group=0.459 Int=0.641
	RET	12.40±11.93	12.60±12.86	
SWLS: (Total Score)	AET	27.75±6.70	32.00±2.58	Time=0.131 Group=0.417 Int=0.281
	RET	26.00±6.36	26.80±8.01	
QOLIBRI: Self (Scale score)	AET	91.96±11.43	89.29±6.52	Time=0.449 Group=0.308 Int=0.224
	RET	73.57±25.70	84.29±16.87	
QOLIBRI: Daily life & Autonomy (Scale score)	AET	87.50±20.31	87.50±18.09	Time=0.689 Group=0.849 Int=0.689
	RET	82.86±18.11	87.86±17.79	
QOLIBRI: Social relationships (Scale score)	AET	94.79±7.89	92.71±14.58	Time=0.591 Group=0.344 Int=0.401
	RET	74.17±32.60	83.33±27.16	
QOLIBRI: Emotions (Scale Score)	AET	92.50±9.57	91.25±8.54	Time=0.339 Group=0.410 Int=0.425
	RET	87.00±13.04	74.00±37.32	
QOLIBRI: Physical (Scale score)	AET	86.25±17.02	87.50±13.23	Time=0.911 Group=0.435 Int=0.911
	RET	76.00±19.81	76.00±28.81	
QOLIBRI: Overall QOL (Scale score)	AET	85.30±13.52	85.64±13.39	Time=0.348 Group=0.572 Int=0.414
	RET	76.72±20.20	81.35±16.87	
TBI-QOL: Physical Health (Index score); n=8	AET	117.50±6.35	114.75±4.50	Time=0.970 Group=0.436 Int=0.402
	RET	107.50±18.38	110.50±16.36	
TBI-QOL: Emotional Health (Index score); n=7	AET	110.50±4.95	113.00±5.66	Time=0.488 Group=0.770 Int=0.964
	RET	114.60±16.83	116.80±17.96	
TBI-QOL: Cognitive Health (Index score)	AET	104.00±16.79	110.75±7.41	Time=0.110 Group=0.856 Int=0.974
	RET	105.80±19.87	112.80±16.57	
TBI-QOL: Social Health (Index score)	AET	109.00±11.66	107.50±9.04	Time=0.584 Group=0.326 Int=0.379
	RET	97.00±8.60	103.20±18.90	
TBI-QOL: Global (Index score); n=6	AET	107±7.07	109.50±2.12	Time=0.327 Group=0.971 Int=0.625
	RET	104.25±15.20	111.25±19.79	

Note: SD = standard deviation; CI = confidence interval; AET= higher intensity aerobic exercise training; RET = lower intensity rapid-resistive exercise training; VO_2=O_2 consumption; RER=respiratory exchange ratio; bpm = beats/min; BVMT-R= Brief Visual Memory Test-Revised; TMT-B=Trail Making Test B; TMT-A=Trail Making Test A; CVLT-II= California Verbal Learning Test; rpm = revolutions per minute; LOS = Limits of Stability; MCT = Motor Control Test; SOT = Sensory Organization Test; FSS = Fatigue Severity Scale; PSQI = Pittsburgh Sleep Quality Index; BDI-II = Beck Depression Inventory-II; NSI = Neurobehavioral Symptom Inventory; SWLS = Satisfaction with Life Scale; QOLIBRI = Quality of Life after Brain Injury; TBI-QOL = TBI-Quality of Life.

Table SVI. Comparison between changes during the exercise intervention period (Exercise; post- minus pre-training) and follow-up period (Follow-up; follow-up minus post-training) for all variables investigated

Outcomes	Exercise (mean± SD)	Follow-up (mean ± SD)	Mean difference (95% CI)	p-Value	Cohen's d
**Cardiorespiratory Fitness (n=4)**					
ΔTime to peak exercise (s)	25.53±65.27	-24.78±8.25	-50.30 (-160.26 to 59.66)	0.241	-0.728
ΔPeak work rate (Watts)	11.75±47.829	-13.00±16.228	-24.75 (-107.06 to 57.56)	0.409	-0.478
ΔRelative VO_2 (ml/min/kg)	0.77±3.42	-0.855±1.13	-1.63 (-8.38 to 5.12)	0.499	-0.384
ΔAbsolute VO_2 (ml/min)	34.65±284.29	-53.01±116.27	-87.66 (-681.88 to 506.56)	0.671	-0.235
ΔRER	-0.028±0.152	-0.033±0.082	-0.01 (-0.37 to 0.36)	0.965	-0.024
ΔHeartrate (bpm)	-4.25±8.461	1.00±4.546	5.25 (-11.66 to 2.27)	0.396	0.494
**Neuropsychological Assessment (n=6)**					
ΔD-KEFS: Correct Sorts (Scale score)	0.00±2.37	1.00±1.67	1.00 (-2.98 to 4.98)	0.547	0.264
ΔD-KEFS: Description (Scale score)	-0.67±2.34	2.00±2.19	2.67 (-1.82 to 7.15)	0.187	0.624
ΔBVMT-R: Immediate Recall (T-score)	9.33±9.63	-7.00±14.44	-16.33 (-41.31 to 8.65)	0.154	-0.686
ΔBVMT-R: Learning (T-score)	-2.00±5.62	-4.50±8.36	-2.50 (-10.81 to 5.81)	0.474	-0.316
ΔBVMT-R: Delayed recall (T-score)	8.50±13.55	-11.33±14.62	-19.83 (-47.26 to 7.59)	0.122	-0.759
ΔTMT-A (T-score)	2.83±13.23	1.50±6.92	-1.33 (-20.79 to 18.13)	0.867	-0.072
ΔTMT-B (T-score)	5.83±9.24	1.00±6.96	-4.83 (-11.93 to 2.27)	0.141	-0.714
ΔCVLT-II: Free Recall (T-score)	1.50±9.65	5.67±7.06	4.17 (-6.61 to 14.94)	0.366	0.406
ΔCVLT-II: Short delay free recall (Standard score)	0.17±1.21	0.92±1.07	0.75 (-1.09 to 2.59)	0.343	0.428
ΔCVLT-II: Long delay free recall (Standard score)	0.58±1.66	0.92±1.07	0.33 (-1.83 to 2.50)	0.709	0.161
**Mobility (n=5, unless otherwise noted)**					
ΔGait Velocity: Regular walking (cm/s)	5.46±15.15	-2.04±4.74	-7.50 (-30.16 to 15.16)	0.410	-0.411

ΔGait Velocity: Fast walking (cm/s)	7.94±28.36	4.90±7.59	-3.04 (-38.37 to 32.29)	0.823	-0.107
Elliptical Cadence: Self-Selected (rpm), n=4	-5.66±9.11	-0.99±8.87	4.67 (-20.16 to 29.50)	0.592	0.299
Elliptical Cadence: Fast (rpm), n=4	9.96±6.16	-4.60±7.92	-14.56 (-35.86 to 6.74)	0.118	-1.088
**Balance (n=5)**					
ΔLOS: Reaction Time (s)	-0.12±0.35	-0.02±0.24	0.10 (-0.62 to 0.81)	0.723	0.170
ΔLOS: Endpoint Excursion (%)	7.20±11.61	4.80±13.08	-2.40 (-27.72 to 22.92)	0.805	-0.118
ΔLOS: Movement Velocity (deg/s)	0.78±0.84	-0.18±0.78	-0.96 (-2.91 to 0.99)	0.244	-0.610
ΔLOS: Maximum Excursion (%)	9.00±11.47	5.20±10.57	-3.80 (-29.31 to 21.71)	0.700	-0.185
ΔLOS: Directional Control (%)	2.80±6.14	4.40±7.23	1.60 (-13.32 to 16.52)	0.781	0.133
ΔMCT: Composite (msec)	4.00±10.07	-24.40±72.01	-28.40 (-124.69 to 67.89)	0.459	-0.366
ΔSOT: Composite	1.20±10.47	0.40±2.88	-0.80 (-16.17 to 14.57)	0.892	-0.065
ΔSOT: Vestibular	3.60±21.87	6.20±13.24	2.60 (-40.52 to 45.72)	0.875	0.075
ΔSOT: Somatosensory	-2.60±3.29	1.00±1.87	3.60 (-0.57 to 7.77)	0.075	1.071
ΔSOT: Visual	0.60±7.16	-0.20±13.10	-0.80 (-17.95 to 16.35)	0.903	-0.058
ΔSOT: Preference	1.20±15.22	0.00±10.17	-1.20 (-29.75 to 27.35)	0.913	-0.052
**Questionnaire (n=6, unless otherwise noted)**					
ΔFSS: Mean (score)	0.1481±1.567	-0.63±1.094	-0.78 (-3.31 to 1.75)	0.465	-0.323
ΔPSQI: Global (score), n= 4	0.40±2.074	0.20±2.168	-1.75 (-5.73 to 2.23)	0.256	-0.700
ΔBDI-II: Total (Score)	-2.33±3.39	2.50±4.81	4.83 (-2.17 to 11.84)	0.136	0.724
ΔBSI-18: Somatization (T-score)	-0.67±2.73	-3.00±7.35	-2.33 (-11.76 to 7.09)	0.553	-0.260
ΔBSI-18: Depression (T-score)	1.17±4.40	-0.50±3.39	-1.67 (-9.59 to 6.26)	0.612	-0.221
ΔBSI-18: Anxiety (T-score)	-3.50±8.78	2.50±8.38	6.00 (-11.06 to 23.06)	0.408	0.369
ΔBSI-18: Global Severity Index (T-score)	-1.33±3.01	-2.33±4.93	-1.00 (-7.64 to 5.64)	0.714	-0.158

ΔNSI: Vestibular (sum score)	-0.17±0.41	0.33±1.37	0.50 (-0.95 to 1.95)	0.415	0.363
ΔNSI: Somatosensory (sum score)	-0.33±1.37	0.50±2.43	0.83 (-2.38 to 4.05)	0.534	0.272
ΔNSI: Cognitive (sum score)	-0.33±0.52	1.17±3.66	1.50 (-2.36 to 5.36)	0.363	0.408
ΔNSI: Affective (sum score)	-1.17±2.23	1.33±3.67	2.50 (-3.46 to 8.46)	0.330	0.440
ΔNSI: Total (sum score)	-2.00±2.97	4.00±10.30	6.00 (-5.67 to 17.67)	0.243	0.540
ΔSWLS: Total (score)	3.33±4.89	-1.67±3.01	-5.00 (-11.77 to 1.77)	0.116	-0.775
ΔQOLIBRI: Self (scaled score)	3.57±15.65	-0.60±6.15	-4.17 (-26.33 to 18.00)	0.649	-0.197
ΔQOLIBRI: Daily life & Autonomy (scaled score)	0.60±3.51	-5.36±11.68	-5.95 (-21.25 to 9.35)	0.363	-0.408
ΔQOLIBRI: Social relationships (scaled score)	0.00±6.97	-0.69±9.65	-0.69 (-16.68 to 15.29)	0.915	-0.046
ΔQOLIBRI: Emotion (scaled score)	0.00±5.48	-2.50±11.29	-2.50 (-17.98 to 12.98)	0.695	-0.170
ΔQOLIBRI: Physical (scaled score)	5.83±8.61	-6.67±11.69	-12.50 (-31.20 to 6.20)	0.146	-0.702
ΔQOLIBRI: Cognitive (scaled score)	8.33±4.88	0.60±11.83	-7.74 (-22.24 to 6.76)	0.228	-0.560
ΔQOLIBRI: Overall (scaled score)	3.15±5.90	-2.36±7.55	-5.52 (-18.63 to 7.59)	0.329	-0.442
ΔTBI-QOL: Physical Health (Index score), n=5	2.00±10.42	3.40±11.97	1.40 (-20.70 to 23.50)	0.869	0.079
ΔTBI-QOL: Emotional Health (Index score), n=4	3.75±7.50	-2.50±4.80	-6.25 (-21.97 to 9.47)	0.295	-0.633
ΔTBI-QOL: Social Health (Index score)	1.67±7.20	2.67±6.56	1.00 (-12.87 to 14.87)	0.860	0.076
ΔTBI-QOL: Cognitive health (Index score)	9.17±11.77	-11.00±15.80	-20.17 (-46.96 to 6.63)	0.111	-0.790
ΔTBI-QOL: Global (Index score), n=4	7.25±7.68	-4.00±10.23	-11.25 (-35.91 to 13.41)	0.243	-0.726
